# Exploring sleep-related problems among concerned others of individuals with mental health problems and/or problematic substance use: a cross-sectional study

**DOI:** 10.3389/fpubh.2026.1703652

**Published:** 2026-03-05

**Authors:** Anita Øgård-Repål, Siri Håvås Haugland, John-Kåre Vederhus, Anja Nastasja Robstad, Tonje Holte Stea

**Affiliations:** 1 Department of Health and Nursing Science, University of Agder, Kristiansand, Norway; 2 Department of Psychosocial Health, University of Agder, Grimstad, Norway; 3 Addiction Unit, Sørlandet Hospital, Kristiansand, Norway

**Keywords:** alcohol use, caregivers, mental health, sleep-related symptoms, substance use

## Abstract

**Background:**

Mental health problems and/or problematic substance use (including alcohol) affect not only the individuals directly involved but also their close family members. Being a concerned other, a partner, parent, or child of someone with mental health problems or problematic substance use can be emotionally demanding and may negatively impact sleep health. This study aimed to examine whether concerned others are at increased risk of sleep-related symptoms compared with individuals without such family-related burdens.

**Materials and methods:**

A cross-sectional study was conducted among 18,517 Norwegian adults (>18 years) by completing an online self-report questionnaire. Multivariable logistic regression models adjusted for age, economic capability (SES) and gender were used to examine whether being a partner or parent of individuals struggling with substance use or mental health problems or having experienced a parent with problematic alcohol use or mental health problems during childhood was associated with sleep-related symptoms.

**Results:**

During childhood, 15.8% and 13.9% reported having parents with mental health problems or problematic alcohol use, respectively. In adulthood, 1 in 6 individuals reported being a partner or parent to someone with mental health problems, and 1 in 10 reported being a partner or parent to someone with problematic substance use. Childhood exposure to parental mental health problems or problematic alcohol use was significantly associated with increased odds of sleep initiation difficulties, sleep maintenance problems, daytime sleepiness, and chronic sleep issues in adulthood (ORs ranging from 1.43 to 1.73). Similarly, being a concerned other to someone with mental health problems or problematic substance use in adulthood was associated with increased odds of having the same sleep-related symptoms (ORs ranging from 1.56 to 1.95).

**Conclusion:**

Concerned others of individuals with mental health problems or problematic alcohol or substance use had higher prevalence of sleep-related symptoms. Both current caregiving and childhood exposure to such experiences are linked to poor sleep and potential long-term health consequences. These findings highlight the need for targeted support and early interventions to improve sleep and overall well-being among concerned others.

## 1 Introduction

The global prevalence of individuals living with mental health disorders or affected by substance use disorders has been estimated to be 970 million and 35 million, respectively (1, 2). Problematic substance use, including alcohol, and/or mental health problems, affects not only individuals but also strains family relationships (1–6). For each individual struggling with problematic substance use, an average of three close family members are directly affected (7). Being a concerned other (family of an individual with a mental health problem and/or substance use problem) is experienced as highly stressful, as it causes uncertainty and worries within a family. Having a family member suffering from alcohol- or substance use disorder/mental health disorder may have cascading effects and lead to adverse health outcomes for the entire household (3, 4). This manifests in various ways, including emotional distress and disrupted family dynamics, all of which contribute to the overall worsening of health and well-being among concerned others (3, 4). Additionally, the concerned others of individuals with problematic substance use often suppress their emotions, take on the responsibility of solving others’ problems, and prioritize others’ needs over their own (8). Moreover, recent research (5, 6, 9) has shown that assuming a role of concerned other is associated with poorer overall, physical, and mental health. These physical and psychological health consequences may, in themselves, contribute to sleep-related symptoms among concerned others, such as problems with initiating and maintaining sleep, and chronic sleep issues.

Research demonstrates that strained family relationships are associated with troubled sleep, and concerned others frequently experience sleep-related symptoms (9, 21, 22). A review by Byun et al. (10) found that sleep problems are common among family caregivers; however, mental health problems or problematic substance use among the care recipients were not included in the study. Although concerned other of individuals with problematic alcohol- or substance use are known to experience significant emotional and physical burden, the specific issue of sleep problems remains underexplored. In their review of caregiver burden measurement related to substance use disorder, Tyo and McCurry (11) analysed 32 studies and identified a wide range of outcomes, including anxiety, depression, emotional distress, and reduced quality of life. However, sleep problems were only mentioned explicitly in one of the included studies (23).

Despite the well-documented role of sleep in maintaining physical and mental health, and evidence from other caregiving contexts highlighting the prevalence and impact of sleep disruptions among caregivers (10), this topic has received little attention in the substance use disorder caregiver literature.

From a public health perspective, identifying risk factors for sleep disorders is crucial, as these conditions are associated with a wide range of comorbid health problems. A recent study by Lee et al. (12) revealed that individuals with insomnia have an increased risk of frailty and chronic diseases, such as cardiovascular disease, diabetes, and cancer, an association that was not observed among weekend catch-up sleepers. Their results suggested that individuals with insomnia have a 72% to 188% greater risk for cardiovascular disease, diabetes, cancer, and frailty, whereas weekend catch-up sleepers do not present an increased risk for these chronic conditions.

This study aims to examine sleep-related symptoms among concerned others of individuals with mental health problems, and/or problematic alcohol or substance use compared with individuals without such family burdens. While our primary focus is on individuals who are currently concerned others, i.e., adults with a partner or child who has mental health problems and/or problematic substance use, we also include those who had such an experience in childhood. Understanding the connection between concerned others’ burdens and concerned others’ sleep-related symptoms would provide valuable insight. These findings underscore the need for awareness and interventions to address these sleep issues, ultimately enhancing the well-being of affected family members. In turn, such support may help them manage their burdens more effectively and contribute to improved family functioning.

## 2 Materials and methods

### 2.1 Study design and population

The present cross-sectional study was part of the Norwegian Counties Public Health Surveys (NCPHS) (13), which were conducted among adult residents (≥18 years of age) living in southern Norway between September and October 2023. The NCPHS is an online health survey that consists of data on health, well-being, childhood, living conditions, local environments, accidents, and injuries among adults aged ≥18 years. Individuals with unverified contact information, addresses outside the included municipalities (not commuters or clients at institutions, etc.), and those without a valid Norwegian birth number or having a temporary residence permit valid for less than 6 months were excluded from the study. A total of 247,698 individuals registered in the Norwegian population register met these eligibility criteria. To enable digital recruitment and communication, individuals who had opted out of digital communication were excluded, as were those lacking unique contact information—specifically, a valid personal telephone number and email address. After these exclusions, the sampling pool was reduced to 217,785 eligible individuals. From this refined population, the NCPHS study team drew a representative sample of 57,891 individuals. This sampling strategy ensured that the recruited participants reflected the demographic composition of the broader adult population in Agder while maintaining practical feasibility for survey administration through digital channels. For individuals who did not complete the survey by the specific deadlines, a total of four reminders were issued: two by email, one by SMS, and an additional SMS reminder for participants under 45 years of age. Written and oral information about the study was provided through webpages and social media (Facebook, official webpages, regional and local newspapers, and radio and television). To increase the participation rate, ten random participants each received a gift card worth NOK 2500 (approximately EUR 214). A total of 18,517 adults agreed to participate (response rate, 32%) by completing an online consent form before completing an online self-report questionnaire. Approximately 15 min were used to complete the study. Participation in the present study was voluntary, and all participants had the opportunity to withdraw from the study at any time and had all personal information deleted.

The Norwegian Institute of Public Health was legally responsible for the NCPHS and ensured the process of collecting and anonymizing data before they were analysed by an independent researcher who had no access to original files containing personally identifiable information.

The present study was conducted in accordance with the Declaration of Helsinki. Ethical approval and research clearance were obtained from the Regional Committee for Medical Research Ethics (file number: 686377).

### 2.2 Measures

All the measures were based on self-reported data, and the questions, response alternatives and variable definitions are presented in the Supplementary file S1.

#### 2.2.1 Concerned other in adulthood

Information about having a child or partner with mental health problems or problematic substance use was retrieved with the following questions: “Are you or have you been partner with or parent to individuals with mental health problems who have affected their daily life?” and “Are you or have you been partner with or parent to individuals with substance use problems/substance use disorders?.” For both questions, the response options were “yes” and “no.”

#### 2.2.2 Concerned other during childhood

The childhood experiences of living with caregivers affected by mental health problems or problematic alcohol use were assessed via two items. The participants were asked *“Did you grow up in a home where at least one of your primary caregivers (parents or stepparents) had mental health problems that affected everyday family life?”* and *“Did you grow up in a home where at least one of your primary caregivers (parents or stepparents) had an alcohol problem?”* The response options for both questions were “yes” or “no.” *Sleep*-*related complaints/symptoms.*

The participants reported the following three sleep-related symptoms: whether they usually had difficulties falling asleep, labelled “sleep initiation problems”; experienced frequent nightly awakenings, labelled “sleep maintenance problems”; and insomnia duration, labelled “chronic sleep problems.” In addition, they were asked about “daytime sleepiness.” The response options for sleep initiation problems, sleep maintenance problems and daytime sleepiness were “never,” “sometimes,” “1–2 times/week,” and “≥3 times/week.” The response options for chronic sleep problems were “Never,” “< 1 month,” “1–2 months,” “3–6 months,” “7–12 months,” and “> 12 months.”

#### 2.2.3 Control variables

Age and gender were utilized as basic socio-demographic data. A subjective measure of economic capability was applied as a proxy for socioeconomic status, measuring how easy or difficult it is for the respondent’s household to make ends meet day-to-day with their total household income. It was coded on a 7-point scale, where 1 represents “very difficult” and 6 represents “very easy.” A 7th option, “Do not know,” was also available. For the analysis, the variable was dichotomized, with responses 1–3 indicating “low economic capability” and responses 4–7 indicating “moderate/high economic capability.” We also conducted a sensitivity analysis where the “do not know” responses for the economic capability variable were treated as missing. This alternative coding did not change the results.

##### 2.2.3.1 Missing data

Missing data were handled using complete-case analysis, as logistic regression includes only observations with complete data on all variables included in the models. No additional methods for handling missing data, such as multiple imputations, were applied. This decision was based on the nature of the variables, which were not derived from scales or composite instruments, and therefore did not allow meaningful imputation based on responses to other items. Overall, the proportion of missing data was very low across the included variables (0.6%–1.1%).

#### 2.2.4 Statistical analyses

Multivariate logistic regression models were used to examine whether being a concerned other to individuals struggling with problematic alcohol or substance use or mental health problems was associated with sleep-related symptoms. Separate logistic regression models were performed for each of the four exposure groups. Each model assessed associations with sleep disturbance outcomes and was adjusted for age, sex, and economic capability. The results are reported as odds ratios (ORs) with 95% confidence intervals (CIs). Data analyses were performed via IBM SPSS Statistics, version 29, and the level of statistical significance was set to *p* < 0.05.

## 3 Results

The average age in the sample was 51.5 years (SD = 16.4). A total of 15.8 and 13.9% of all the participants reported having had parents with mental health problems or problematic alcohol use, respectively, during childhood. Currently, 1 in 6 individuals reported either being a partner of someone or having a child with mental health problems, and 1 in 10 reported a similar connection to someone with problematic substance use (Table 1). The descriptive analyses are described in Table 1. Categorical variables are presented as frequencies and percentages.

**Table 1:** Descriptive statistics, Norwegian Counties Public Health Survey (Agder 2023).

Characteristics	*n*	%
Male	8,201	44.3
Female	10,316	55.7
Low economic capability	4,739	26.2
Parental mental health problems during childhood	2,893	15.8
Problematic alcohol use during childhood	2,556	13.9
Being partner with or parent to individuals with mental health problems	3,014	16.4
Being partner with or parent to individuals with problematic substance use	1873	10.2

The results presented in Table 2 show that having parents with mental health problems or problematic alcohol use during childhood was associated with increased odds of experiencing sleep-related symptoms related to sleep initiation (OR: 1.60; 95% CI: 1.44–1.77 and 1.61; 1.44–1.79), sleep maintenance (1.43; 1.31–1.56 and 1.45; 1.32–1.58), daytime sleepiness (1.71; 1.56–1.86 and 1.44; 1.31–1.58) and chronic sleep-related symptoms (1.73; 1.59–1.88 and 1.58; 1.45–1.73) in adulthood compared with those who were not concerned other during childhood. Furthermore, the results show that having a child or partner with mental health problems or problematic substance use in adulthood was associated with increased odds of challenges related to sleep initiation (1.74; 1.57–1.92 and 1.95; 1.73–2.19), sleep maintenance (1.57; 1.44–1.70 and 1.56; 1.41–1.73), daytime sleepiness (1.90; 1.75–2.08 and 1.65; 1.48–1.83) and chronic sleep-related symptoms (1.88; 1.73–2.04 and 1.74; 1.57–1.92) compared with those who were not concerned other in adulthood.

**Table 2:** Multivariable logistic regression analysis of sleep indicators by childhood exposure to parental mental health/alcohol problems and current concerned other (CO) status indicators.

Concerned others	Sleep initiation	Sleep maintenance	Daytime sleepiness	Chronic sleep-problems		
	OR (CI 95%)	OR (CI 95%)	OR (CI 95%)	OR (CI 95%)
M 1	Having had parents with mental health problems (in childhood)	1.60 (1.44–1.77)*	1.43 (1.31–1.56)*	1.71 (1.56–1.86)*	1.73 (1.59–1.88)*
M 2	Having had parents with problematic alcohol use (in childhood)	1.61 (1.44–1.79)*	1.45 (1.32–1.58)*	1.44 (1.31–1.58)*	1.58 (1.45–1.73)*
M 3	Having a child or partner with mental health problems (adulthood)	1.74 (1.57–1.92)*	1.57 (1.44–1.70)*	1.90 (1.75–2.08)*	1.88 (1.73–2.04)*
M 4	Having child or partner with problematic substance use (adulthood)	1.95 (1.73–2.19)*	1.56 (1.41–1.73)*	1.65 (1.48–1.83)*	1.74 (1.57–1.92)*

Data are presented as odds ratios (ORs) with 95% confidence intervals (CIs).*p < 0.001.

All the models (M1–M4) were adjusted for age, gender and economic capability (SES).

## 4 Discussion

Findings in the present study indicated a consistent pattern in which individuals who were concerned others for a child or partner reported higher levels of various sleep-related symptoms compared with those who were not. These difficulties included problems with sleep initiation, sleep maintenance, daytime sleepiness, and chronic sleep issues. Similarly, individuals who had grown up with a parent experiencing mental health problems or problematic alcohol use presented comparable patterns of increased risk.

The concerned others in this study reported a broad range of sleep-related symptoms, and our findings indicate that sleep-related symptoms are more prevalent among concerned others of children or partners with mental health problems or problematic substance use than among a reference group of individuals without such family burdens. This heightened vulnerability to sleep-related problems may stem from the emotional and practical demands placed on concerned others in close caregiving roles and are likely to contribute to adverse health experiences and long-term health consequences. These findings are in line with results from other studies showing that strained family relationships, especially those involving substance use disorder/mental health disorder, contribute to sleep-related symptoms among adult concerned others (9). According to a systematic review by Byun et al. (10), approximately 76% of concerned others reported poor sleep quality. Suboptimal sleep, such as insomnia and nappers, can trigger a cascade of negative physical and mental health outcomes (12), thereby impacting numerous aspects of life (14). Overall, our study adds to the growing body of literature on how concerned others are affected by the mental health problems and problematic substance use/problematic alcohol use of close family members (3, 4, 9). A study by Lee et al. (12) suggested that sleep-related symptoms can initially arise as a reaction that, over time, may lead to disease. The results thereby suggest a heightened risk of chronic conditions in those with suboptimal sleep health, particularly those who have insomnia. These findings underscore the importance of recognizing the diversity of concerned other experiences and the need to consider both the concerned other’s relationship with the affected individual and the nature of the individual’s challenges.

Paying attention to the characteristics of concerned others, including their mental and physical health and symptoms such as depression, fatigue, and anxiety, may help identify those most at risk of sleep-related symptoms and guide the development of more targeted support strategies. In light of our findings, it is important not only to assess mental and physical health issues but also to include sleep as a relevant factor influencing overall health outcomes. A deeper understanding of how being a concerned other to someone with problematic substance/alcohol use and/or mental health problems affects concerned others could inform the development of tailored interventions that address these challenges and support the well-being of all family members (9).

Importantly, the results also revealed that those who experienced parental mental health problems or problematic alcohol use during childhood reported moresleep-related symptoms in adulthood. Given that sleep difficulties are associated with adverse physical and mental health outcomes (12, 15), these findings underscore the long-term impact of adverse childhood experiences on health, which is comparable to the effects of current caregiving responsibilities. This aligns with previous research emphasizing the widespread and often overlooked burden experienced by concerned others (3, 4, 20).

The results suggest that individuals who were concerned others in childhood, those exposed to parental mental health problems or problematic alcohol use, may continue to experience sleep-related symptoms into adulthood, even in the absence of current caregiving responsibilities. This finding supports previous research linking adverse childhood experiences to long-term sleep-related symptoms (16) and highlights the importance of addressing both past and present family-related stressors. The connection between sleep habits and health status is likely bidirectional: inadequate sleep can exacerbate health issues, whereas health problems can negatively affect sleep quality (17). Furthermore, individuals with poor sleep quality are more likely to be less physically active, be less involved in daily activities, and have challenges related to maintaining self-control and having anxiety/depression (15). Thus, residing in such circumstances may perpetuate a vicious cycle, where poor sleep quality adversely impacts one’s health and may impair the capacity to maintain self-care and provide for those in one’s immediate environment. The results from the present study underscore the importance of implementing early intervention strategies for at-risk children, as exposure to parental mental health problems or problematic alcohol use during childhood occurred in a notable proportion of cases (15.8% and 13.9%, respectively). By addressing both childhood and adult caregiving experiences, such interventions can be more comprehensive and effective in reducing the long-term burden of concerned others. By focusing on proactive measures for children in affected families, we can work towards reducing long-term suffering and providing socioeconomic benefits through improved health and productivity outcomes (9).

Future research should explore how sleep-related symptoms contribute to cascading effects on physical and mental health due to sleep-related symptoms, including their role in mediating the link between caregiving and health. Interventions aimed at improving sleep and resilience among concerned others should also be evaluated.

### 4.1 Methodological considerations

A strength of this study is the large, randomly selected sample from the general population. This allows for a knowledge contribution that is not solely based on clinical data and includes a broad age range. The sample was, however, somewhat skewed toward participants with higher education. Furthermore, sample characteristics showed a lower participation rate among men and the lowest and highest age groups compared to women and other age groups, which necessitates awareness of potential selection bias. Lack of information about mental health problems among concerned others also limited the interpretation of results. In addition, the study’s cross-sectional design, limit the ability to draw conclusions about causality. Furthermore, s sleep-related complaints/symptoms were assessed using self-reported single items rather than standardized clinical instruments. Childhood exposure was restricted to parental problematic alcohol use, and the survey did not include the potential impact of other substance use disorders. Although heterogeneity in concerned others’ experiences was partly addressed by defining four distinct concerned other groups, some heterogeneity remained, as partner and parent relationships could not be distinguished within adult concerned other categories. A key strength is that the study captures concerned others’ experiences across childhood and adulthood and across different family relationships related to mental health and substance use. The study relies on self-reports, including subjective retrospective accounts. For the exposure variables, this means that older participants were required to recall events that occurred a long time ago. While subjective reports typically show only moderate correlations with more objective measures (18), retrospective self-reports of childhood adversities have been found to be more strongly associated with psychopathology than objective records are, highlighting the relevance of these measures (19). We were not able to rule out the possibility that some participants were still caring for parents with ongoing mental health problems or problematic substance use. Although the overall level of missing data was very low, we cannot be certain that missing was completely at random. Some of the variables addressed sensitive topics, which may have influenced response patterns and introduced potential bias. This should be considered when interpreting the results.

## 5 Conclusion

This study demonstrated that concerned others, both those currently caring for a partner or child with mental health problems or problematic substance use and those who experienced mental health problems or problematic alcohol use among their parents in childhood, are at significantly greater risk of sleep-related symptoms. These include difficulty falling asleep, staying asleep, and experiencing restorative rest, which can negatively affect both physical and mental health and overall functioning.

The findings emphasize how both current caregiving stress and early-life exposure to parental mental health problems or problematic alcohol use contribute to long-term health challenges. Concerned others form a diverse group, and future efforts should consider variations in experiences, needs, and resilience. Thus, these findings suggest that sleep-related symptoms may serve both as a symptom of ongoing stress and as a marker of potential long-term health concerns, including chronic illness and reduced quality of life, and broader societal consequences.

Given the strong link between sleep and overall well-being, there is a clear need for increased awareness and tailored interventions. Support strategies should consider concerned others’ caregiving role, personal health, and past experiences. Early preventive efforts targeting children in affected families are particularly important to reduce long-term consequences and break the cycle of burden across generations. Supporting concerned others is essential not only for their own health but also for the well-being of the families they support.

## Data Availability

The raw data supporting the conclusions of this article will be made available by the authors, without undue reservation.
