# Supplementary material for: Exploring sleep-related problems among concerned others of individuals with mental health problems and/or problematic substance use: a cross-sectional study
Source: Front Public Health. 2026 Mar 5;14:1703652. doi: 10.3389/fpubh.2026.1703652 (PMC13001646; doi:10.3389/fpubh.2026.1703652)
Supplement: Supplementary file 1 [file Table_1.docx]

Supplementary Material

1 Supplementary Data

**Questions, response alternatives and variable definitions**

| **Questions** | **Response Alternatives** | **Variable definition** |
| --- | --- | --- |
| Did you grow up in a home where at least one of your primary caregivers (parents or stepparents) had mental health problems that affected everyday family life? | Yes, no |  |
| Did you grow up in a home where at least one of your primary caregivers (parents or stepparents) had an alcohol problem? | Yes, no |  |
| Are you or have you been partner with or parent to individuals with mental health problems that have affected your daily life? | Yes, no |  |
| Are you or have you been partner with or parent to individuals with substance use problems/substance use disorders? | Yes, no |  |
| **Sleep disturbances** | | |
| How often do you have difficulties falling asleep? | Never/seldom, sometimes, 1-2 times/week, ≥3 times/week | Disturbance related to sleep initiation was defined as ≥3 times per week |
| How often do you experience frequent nightly awakenings? | Never/seldom, sometimes, 1-2 times/week, ≥3 times/week | Disturbance related to sleep maintenance was defined as ≥3 times per week |
| How often do you experience daytime sleepiness? | Never/seldom, sometimes, 1-2 times/week, ≥3 times/week | Disturbance related to daytime sleepiness was defined as ≥3 times per week |
| For how long have you experienced sleep problems? | Never, < one month, 1-2 months, 3-6 months, 7-12 month, > 12 months | Chronic sleep-problems was defined as ≥3 months |
| Financial capability (SES)  For one-person households. Consider your total income. If you live with others, consider the total income of everyone in the household. How easy or difficult is it for you to make ends meet day to day with this income? | 1. Very difficult 2. Difficult 3. Relatively difficult 4. Relatively easy 5. Easy 6. Very easy 7. Don’t know | 1-3 = low economic capability vs.  4-7 = middle/high economic capability |
